# Effects of pesticides on soil microbial community structure and nitrogen transformation in tobacco fields affected by root rot

**DOI:** 10.3389/fmicb.2025.1733977

**Published:** 2026-01-05

**Authors:** Fengyu Li, Zhaoguo Qiu, Zhouyang Pei, Qifa Zhu, Sideng Shen, Linlin Fan, Lvqin Xu, Changquan Huang, Jie Wang, Bin Huang, Leye Huang, Xinyu Liu, Qingli Han

**Affiliations:** 1 College of Forestry, Southwest Forestry University, Kunming, China; 2 Pest Integrated Management Key Laboratory of China Tobacco, Tobacco Research Institute of Chinese Academy of Agricultural Sciences, Qingdao, China; 3 Xuancheng Modern Agricultural Industrial Park, Xuancheng, China; 4 Syngenta (China) Investment Co., Ltd., Shanghai, China

**Keywords:** pesticides, microbial community, xenobiotic degradation, nitrogen cycle gene, co-occurrence network patterns

## Abstract

**Introduction:**

In tobacco planting soil infected with root rot disease, the potential impacts of prothioconazole (T1), pyrisoxazole (T2), kasugamycin combined with *Paenibacillus polymyxa* (T3), and cyclobutrifluram (T4) on soil microecology remain unclear. This study examined their effects on soil microbial communities and nitrogen transformation processes.

**Methods:**

By measuring soil nitrogen forms and enzyme activities, combined with metagenomic sequencing, we conducted a comprehensive assessment of the soil microecology, focusing on shifts in microbial community composition, xenobiotic degradation potential, and nitrogen cycling processes.

**Results and discussion:**

The results revealed that pesticide application significantly changed the content of nitrogen forms and their transformation rate. T1 and T2 treatments significantly increased the accumulation of ammonium nitrogen (NH_4_^+^-N), while T2 and T4 markedly promoted the accumulation of nitrate nitrogen (NO_3_^–^-N). Microbial community analysis indicated that the T2 and T4 treatments significantly affected the microbial structure. Analysis of xenobiotic degradation pathways showed that multiple pathways were suppressed by the four pesticide treatments, with the T2 treatment exhibiting the broadest suppressive effect. Metagenomic analysis further revealed that the T2 treatment promoted the accumulation of both NH_4_^+^-N and NO_3_^–^-N by up-regulating the mineralization gene (*gdh*) and nitrification genes (*hao* and *nxrAB*), while the T4 treatment facilitated NO_3_^–^-N accumulation by up-regulating nitrification genes (*hao* and *nxrAB*). Correlation network analysis uncovered relationships between key nitrogen cycle genes and microbial genera, showing that nitrification genes (*hao* and *nxrAB*) in the T2 and T4 treatment groups exhibited positive correlations with *Nitrobacter* and *Nitrosovibrio*. This research clarifies the pathways through which these four pesticides influence the soil nitrogen cycle, providing an important theoretical basis for their ecological risk assessment and rational application.

## 1 Introduction

Root rot caused by soil-borne pathogens often occurs in combination with bacterial wilt and tobacco black shank disease, severely limiting tobacco yield and quality (Zhang G. et al., 2025). The application of pesticides serves as one of the most effective measures to control disease by suppressing pathogenic microbial, thereby reducing crop losses and ensuring agricultural output (Tang et al., 2023). Studies have shown that various chemical pesticides exhibit control efficacy against tobacco black root rot (Chen et al., 2022; Liu et al., 2025).

However, the application of pesticides not only targets pathogens but also disturbs the soil microbiota. Such disturbances can lead to substantial changes in soil community composition and key ecosystem processes (Han et al., 2024; Wang Y. et al., 2025). As the core component maintaining soil health and ecosystem stability, microbial communities extensively participate in and regulate key nitrogen cycle processes such as nitrification, denitrification, and nitrogen fixation. These processes directly influence soil nitrogen supply capacity and crop nutrient use efficiency (Kuypers et al., 2018; Trivedi et al., 2020). Of particular concern is that pesticide can alter the soil microbial community structure and disrupt nitrogen transformation functions (Hou et al., 2024).

Among the currently used pesticides, prothioconazole, a triazole fungicide, has a half-life of less than 5.82 days (Lin et al., 2017; Gao et al., 2020). Studies have indicated that it can significantly affect soil microbial communities and markedly inhibit the nitrification process (Zhai et al., 2022). Pyrisoxazole, an isoxazoline-class fungicide (Jiao et al., 2022), has half-lives of 2.4–8.4 days in vegetables, 7.4–10.3 days in fruits, and 8.2–100.4 days in soil (Pan et al., 2016; Qi et al., 2016; Yang et al., 2017). Both kasugamycin and Polymyxin are antibiotics, which often significantly impact soil microbial communities (Kong et al., 2025; Materon and Palzkill, 2023; Cycoń et al., 2019). Cyclobutrifluram is a new-generation succinate dehydrogenase inhibitor fungicide (Zhang et al., 2024). Although the ecological effects of prothioconazole have received some attention, the impacts of the other three pesticides on the microbial community structure and nitrogen cycling functions in tobacco root rot field soils remain poorly understood, and their ecological behavior and effects have yet to be fully elucidated.

To compare the non-target effects of pesticides with diverse mechanisms, this study assessed how prothioconazole, pyrisoxazole, kasugamycin combined with *Paenibacillus polymyxa*, and cyclobutrifluram impact soil in tobacco fields affected by root rot. That is (1) The changes in soil nitrogen content and enzyme activities; (2) Changes in soil microbial community diversity and structure; (3) Changes in the soil’s capacity for xenobiotic biodegradation; (4) The abundance of nitrogen cycle genes and their correlations with the microbial community. This research provides a systematic assessment of the impacts of these four pesticides on soil physicochemical properties, microbial communities, and nitrogen cycle genes, which is of significant importance.

## 2 Materials and methods

### 2.1 Field experiment design and sample collection

The field experiment was located at the Southern Anhui Tobacco Experiment Station of the Chinese Academy of Agricultural Sciences in Xuancheng City, Anhui Province (118°45′E, 30°56′N). The regional climate is classified as subtropical monsoon, characterized by mild, humid conditions with precipitation concentrated in the warm season. Experiments were conducted in local farmlands with flat terrain and stable soil. Critically, the field had a confirmed history of tobacco root rot, ensuring natural pathogen pressure for the experiment; the land was fallow before the current trial. Before establishing the experimental treatments, initial soil sampling was conducted to assess the field’s spatial homogeneity. Three independent composite samples were collected from across the field using the five-point sampling method, with soil taken from a depth of 0–20 cm after clearing surface debris. These samples were air-dried, homogenized, and analyzed separately, confirming a consistent baseline for the subsequent experiment.

The experiment included four pesticide treatments and a control, with all application concentrations selected based on the manufacturers’ recommended field application rates: T1: prothioconazole (250 g/L EC, 4400× dilution, Shandong Qingdao Kaiyuanxiang Chemical Co., Ltd.); T2: pyrisoxazole (25% EW, 2200×, Jiangsu Yangnong Chemical Co., Ltd.); T3: kasugamycin combined with *Paenibacillus polymyxa*, (3% SC, 1800×, Wuhan Kernel Bio-tech Co, Ltd.); T4: cyclobutrifluram (450 g/L SC, 6000×, Syngenta Crop Protection Co., Ltd.). A randomized complete block design featuring three replicates was implemented, accommodating a total of 15 individual plots, each covering an area of 100 m^2^. Tobacco was transplanted at a density of 18,000 plants per hectare with a row spacing of 1.2 m and plant spacing of 0.5 m, yielding an aboveground dry biomass of approximately 2,470 kg per hectare at harvest. All plots received identical management except for pesticide application. Pesticides were applied via root irrigation at transplanting and again 20 days later, with 200 ml of diluted solution per plant per application. An equivalent volume of water was applied to the CK group in place of the pesticide solutions.

Two months after tobacco transplanting, soil samples were obtained from every treatment plot via the five-point method. After removing surface debris such as leaves, weeds, and gravel, Sampling was conducted at a depth of 0–20 cm with alcohol-sterilized samplers, and approximately 0.3 kg of soil was obtained from each sampling point. Concurrently, relevant information including plot number and sampling date was recorded. Soil from each plot was thoroughly mixed and divided into two aliquots. A 0.5 kg aliquot was snap-frozen in liquid nitrogen for microbial analysis, while a 1 kg aliquot was air-dried for physicochemical analysis.

### 2.2 Soil nitrogen content and enzyme activities measuring

NH_4_^+^-N and NO_3_^–^-N from soil are extracted using potassium chloride solution (Saha et al., 2018). NH_4_^+^-N reacts with phenol to form blue indophenol dye, while NO_3_^–^-N reacts with N(1-naphthyl) -ethylenediamine hydrochloride to produce red dye. Soil pH was determined potentiometrically using a 1:5 (w/v) soil-water suspension (Faria et al., 2023). The soil moisture content is calculated based on the quality difference by using the drying method (Astm International, 2010). Soil bulk density was determined by the ring knife method (Jia et al., 2025). A certain volume of unprocessed soil was collected, dried and weighed. Soil organic matter was detected by the potassium dichromate oxidation method (Xu et al., 2025). The activities of urease, dehydrogenase, nitrate reductase, and nitrite reductase were evaluated with commercial assay kits (Beijing Boxbio Science & Technology Co., Ltd.), in accordance with the manufacturer’s instructions (Page, 1982).

### 2.3 Metagenomic sequencing

Metagenomic DNA was isolated from soil samples using the E.Z.N.A.^®^ Soil DNA Kit (Omega Bio-tek, USA). Following paired-end sequencing on an Illumina NovaSeq platform (Majorbio, China), raw sequencing data underwent quality control with FASTP (v 0.20.0). High-quality reads were subsequently assembled into contigs via MEGAHIT (v 1.1.2) (Li et al., 2015). Contigs meeting a length threshold of ≥ 300 bp were retained for subsequent gene prediction using MetaGene (Noguchi et al., 2006). A non-redundant gene set was then constructed with CD-HIT (v 4.6.1) (Fu et al., 2012), applying thresholds of 90% for both sequence identity and coverage. For taxonomic classification, the representative sequences from this gene set were aligned to the NCBI NR database using DIAMOND (v 0.8.35) (Buchfink et al., 2015) with a maximum e-value of 1e-5. Furthermore, nitrogen cycle-related genes were identified through KEGG annotation, also performed with DIAMOND (v 0.8.35) (Pal et al., 2014; Metch et al., 2018). All obtained raw sequence datasets have been uploaded to the NCBI Sequence Read Archive (SRA) with the accession number PRJNA1346056.

### 2.4 Data analysis

Microbial community composition was analyzed using R (v3.3.1). Specifically, at the phylum level, the prevalent microbial communities were profiled using bar plots. The overall microbial structure was assessed through Principal Coordinates Analysis (PCoA) based on Bray-Curtis distances. To determine the significance of differences in community structure, Permutational Multivariate Analysis of Variance (PERMANOVA) was performed with 999 permutations (Ezeokoli et al., 2020). Alpha diversity were estimated with the mothur package (v1.30.2) (Schloss et al., 2009), as measured by the Shannon–Wiener index {H′ = −Σ[p_i × ln(p_i)]} (Shannon, 1948) and Chao1 richness estimator [Chao1 = S_obs + (F_1_^2^/2F_2_)] (Chao, 1984). LEfSe (Linear Discriminant Analysis Effect Size) was employed to identify differentially abundant taxa, discerning features and associated categories that exhibited significant differences. Xenobiotic biodegradation pathways were annotated using the Tax4Fun software (Aßhauer et al., 2015) with reference to the KEGG pathway database. Network analysis was based on Spearman correlation analysis (*p* < 0.05, *r* = 0.5) on Gephi platform software (Huang et al., 2021). Key genes involved in nitrogen cycle processes were identified by querying the nitrogen metabolism pathway within the KEGG database. A schematic diagram of the nitrogen cycle metabolic pathway was created using Adobe Illustrator. SPSS 23.0 software was employed to conduct the statistical analysis. To assess statistical significance, differences among samples were evaluated by one-way ANOVA followed by Duncan’s multiple range test.

## 3 Results

### 3.1 Changes in soil physicochemical properties and enzyme activities

This study assessed the impact of four pesticide treatments on the concentrations of NH_4_^+^-N and NO_3_^–^-N, alongside the activities of four key soil enzymes: urease, dehydrogenase, nitrate reductase, and nitrite reductase. Before the start of the experimental treatment, the basic soil properties of the entire experimental field were determined. The soil was weakly acidic (pH 6.16 ± 0.12), with moderate organic matter content (25.28 ± 2.80 g/kg) and suitable physical structure (bulk density 1.14 ± 0.09 g/cm^3^, water content 14.17 ± 0.31%). The coefficients of variation for key physicochemical parameters among the three sampling sites were relatively low (e.g., 1.9% for pH and 2.2% for water content), providing direct evidence of the initial homogeneity of the entire experimental field.

**FIGURE 1:**
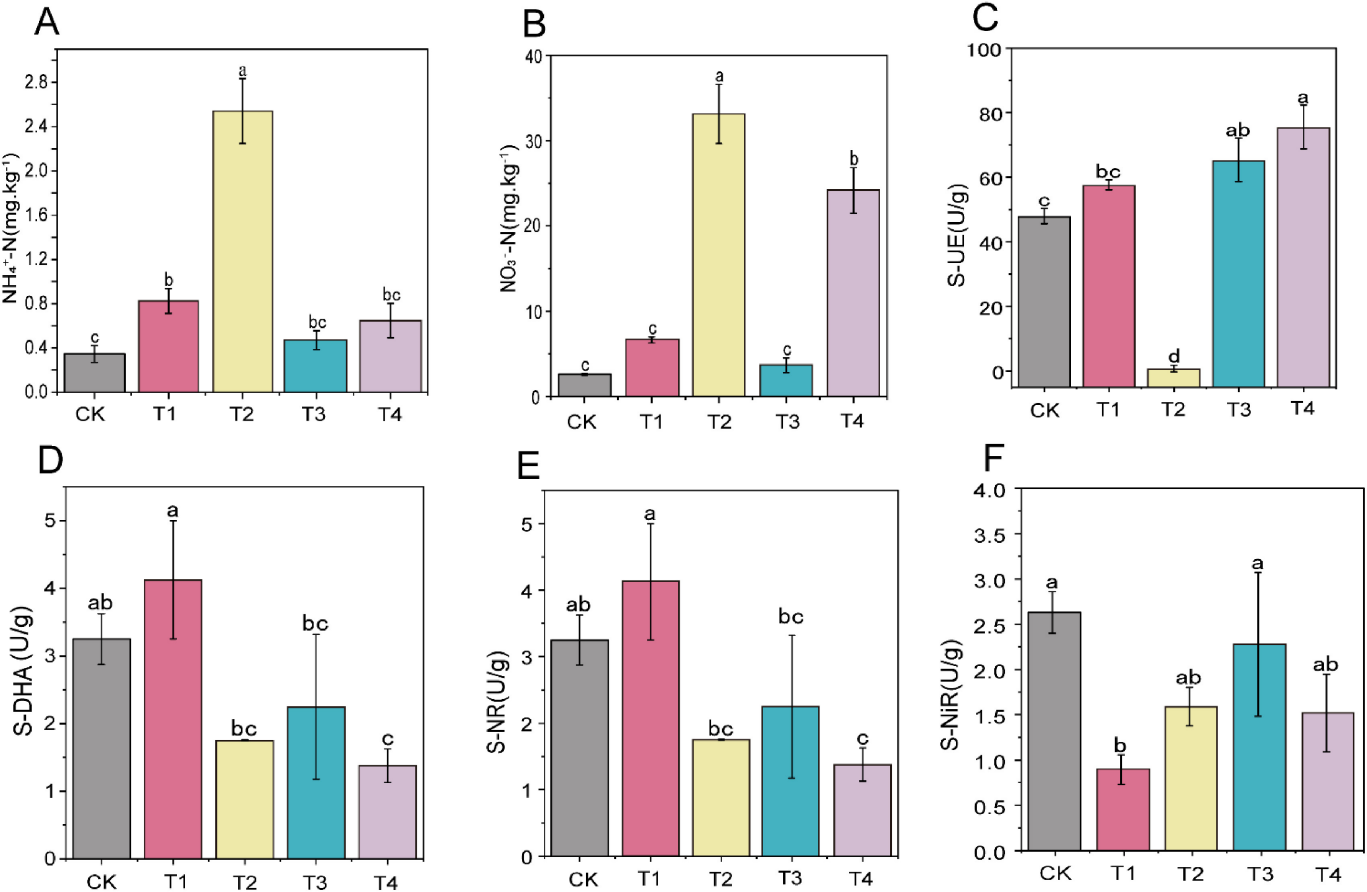
Changes of soil nitrogen content and enzyme activities. **(A)** Ammonium nitrogen (NH_4_^+^-N); **(B)** nitrate nitrogen (NO_3_^–^-N); **(C)** soil urease (S-UE); **(D)** soil dehydrogenase (S-DHA); **(E)** soil nitrate reductase (S-NR); **(F)** soil nitrite reductase (S-NiR). The superscript letters ‘a–c’ indicate statistically significant differences (*p* < 0.05) among different treatments as determined by Duncan’s multiple range test.

Pesticide treatments significantly affected (*P* < 0.05) soil NH_4_^+^-N and NO_3_^–^-N. As illustrated in Figures 1A, B, T1 and T2 markedly increased the soil NH_4_^+^-N content by 2.38 times and 7.34 times, respectively. After T2 and cyclobutrifluram (T4) treatments, the soil NO_3_^–^N content increased by 12.83 times and 9.36 times (*P* < 0.05), respectively. As shown in Figures 1C–F, soil urease activity was significantly increased by 1.36 times in the T3 (kasugamycin combined with *Paenibacillus polymyxa*) treatment and by 1.58 times in T4, whereas the T2 treatment resulted in significant inhibition (Figure 1C). After the T4 treatment, soil dehydrogenase and nitrate reductase activities were significantly reduced by 2.36 times and 2.03 times, respectively (Figures 1D, E).

### 3.2 Changes in soil microbial diversity

**FIGURE 2:**
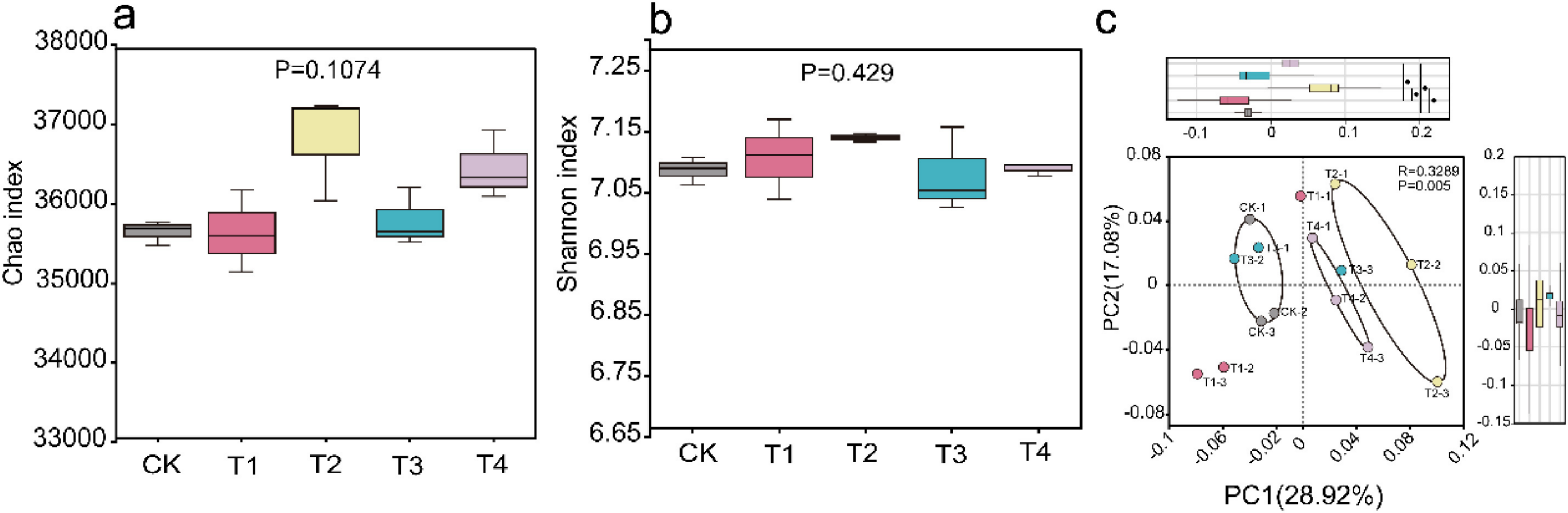
**(A, B)** Boxplot of alpha diversity index differences; **(C)** principal coordinate analysis.

The results of species-level alpha diversity analysis showed no significant effect of pesticide application on the richness or diversity of soil microbial communities. Both the Chao1 and Shannon indices revealed no statistically significant differences between the CK group and the four pesticide treatment groups (Figures 2A, B). The PCOA plot, generated from Bray-Curtis distances, revealed that the variance explained by PC1 and PC2 was 28.92% and 17.08%, respectively. The PCoA results revealed a clear separation among the T2, T4 group, and the CK group, suggesting that the root irrigation treatment of T2 and T4 significantly altered the soil microbial community structure (Figure 2C).

### 3.3 Changes in soil microbial community structure

**FIGURE 3:**
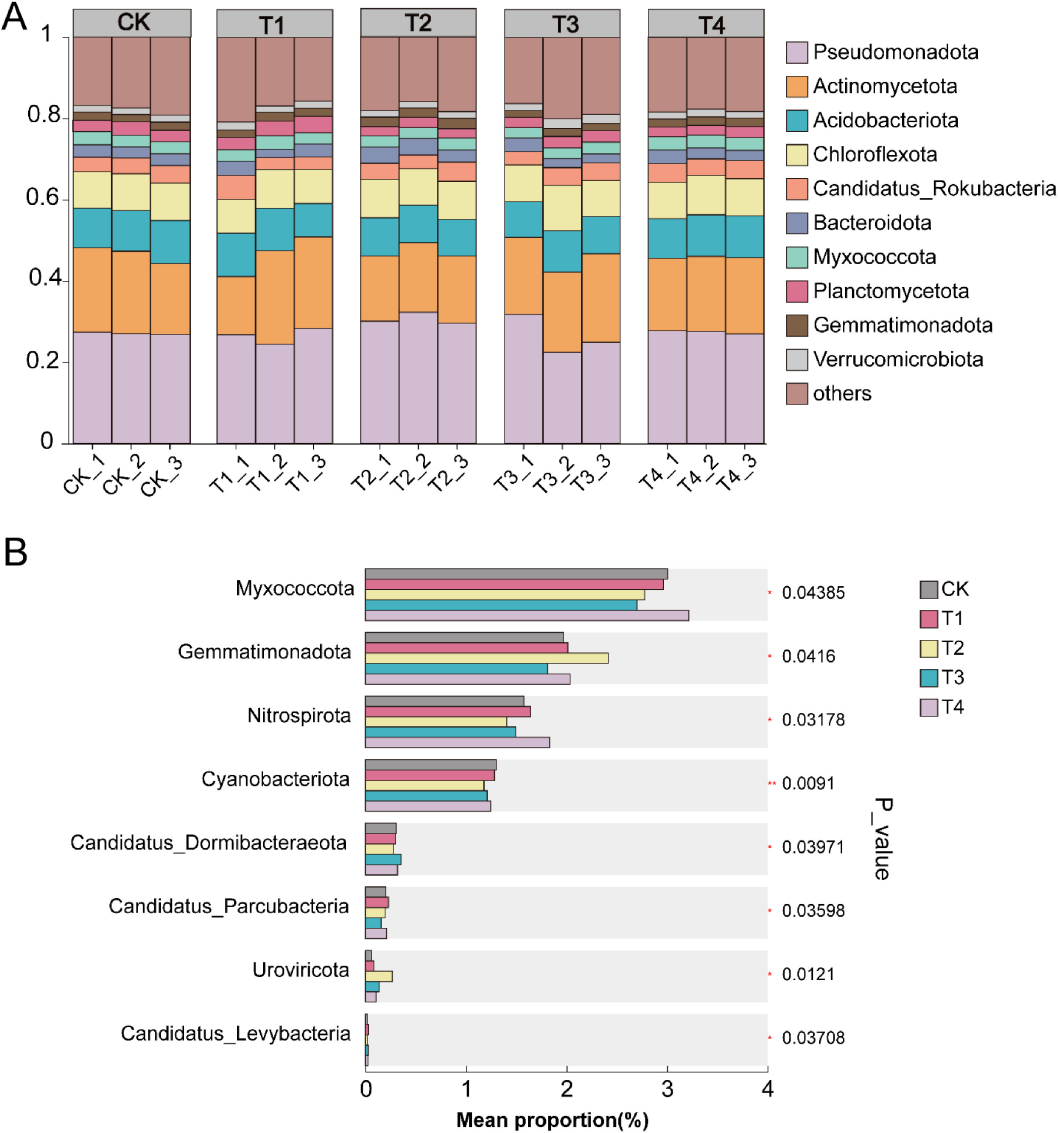
**(A)** Phylum-level profiles of the microbial community; **(B)** significantly different taxa at the phylum level.

Metagenomic sequencing was employed to assess the impact of pesticide application on soil microbial community structure. The dominant phyla across all groups were Pseudomonadota (with proportions of 27.09, 26.55, 31.63, 27.10, and 27.65% in CK, T1, T2, T3, and T4, respectively), Actinomycetota (19.86, 20.26, 16.37, 20.13, and 18.42%), and Chloroflexota (9.24, 8.81, 9.42, 9.77, and 9.43%) (Figure 3A). In comparison to the CK group, Pseudomonadota exhibited an increased relative abundance in the T2, T3, and T4 treatments following pesticide application, while a decrease was observed in the T1 treatment (Figure 3A).

Analysis of significant differences at the phylum level showed that, among the top 10 most abundant phyla, Myxococcota and Gemmatimonadota exhibited significant variations across treatments (Figure 3B). The T2 treatment significantly increased the abundance of Myxococcota, while the T4 treatment group significantly reduced the abundance of Myxococcota. Both T2 and T4 treatment groups significantly increased the abundance of Gemmatimonadota. Actinomycetota was higher in the T1 and T3 treatments but decreased in T2 and T4 (Figure 3A). The relative abundance of Chloroflexota decreased in the T1 treatment but increased in T2, T3, and T4 (Figure 3B).

**FIGURE 4:**
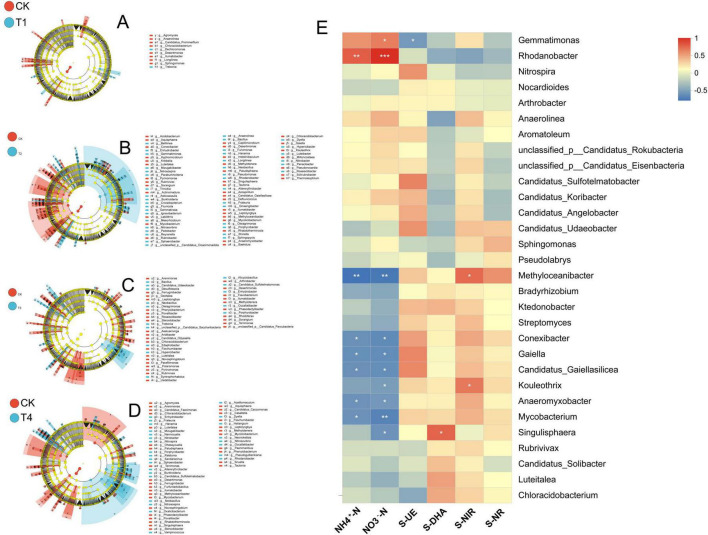
Microbial phylogeny in soils under different pesticide root irrigations: **(A)** prothioconazole; **(B)** pyrisoxazole; **(C)** kasugamycin combined with *Paenibacillus polymyxa*; **(D)** cyclobutrifluram. The diagram displays taxonomic levels from domain to genus as concentric circles. Abundance is indicated by circle diameter. **(E)** Heatmap of the correlation between physicochemical properties and the top 30 abundant microbial genera. Asterisks within heatmap cells indicate the significance level of the Spearman correlation: *p < 0.05, **p < 0.01, and ***p < 0.001.

LEfSe analysis revealed that the T1 treatment group exhibited significant enrichment of the genera *Dechloromonas* and *Trebonia* (Figure 4A). The T2 treatment group showed significant enrichment of *Bellilinea*, *Enhydrobacter*, *Gemmatimonas*, *Mizugakiibacter*, *Nitrosospira*, *Paraburkholderia*, *Trinickia*, *Asticcacaulis*, *Burkholderia*, *Croceibacterium*, *Fluviicola*, *Gemmatirosa*, *Ignavibacterium*, *Mesorhizobium*, *Nitrosovibrio*, *Pedobacter*, *Reyranella*, *Sphaerobacter*, *Anaerolinea*, *Bacillus*, *Fulvimonas*, *Hanamia*, *Longilinea*, *Neobacillus*, *Pseudomonas*, *Rhodanobacter*, *Dyella*, *Hypericibacter*, *Luteibacter*, *Nitrobacter*, *Panacibacter*, and *Roseisolibacter* (Figure 4B). The T3 treatment group demonstrated significant aggregation of *Bacillus*, *Candidatus Udaeobacter*, *Desulfobacca*, *Neobacillus*, *Oleiagrimonas*, *Trebonia*, *Edaphobacter*, *Hypericibacter*, *Syntrophorhabdus*, *Alicyclobacillus*, *Candidatus Sulfotelmatomonas*, *Enhydrobacter*, *Occallatibacter*, and *Porphyrobacter* (Figure 4C). The T4 treatment group exhibited significant enrichment of *Enhydrobacter*, *Frateuria*, *Hanamia*, *Mizugakiibacter*, *Nannocystis*, *Nitrobacter*, *Nitrospira*, *Oxalicibacterium*, *Vampirococcus*, *Acetitomaculum*, *Dyella*, *Haliangium*, *Neorickettsia*, *Nitrosovibrio*, *Occallatibacter*, *Pseudogulbenkiania*, and *Rhodanobacter* (Figure 4D).

Calculation of Spearman correlations between environmental factors and microbial genera showed that the abundance of 5 genera was significantly positively correlated with physicochemical properties, while the abundance of 9 genera was significantly negatively correlated with soil physicochemical factors. NH_4_^+^-N was positively correlated with the genus *Rhodanobacter* and negatively correlated with *Methyloceanibacter*, *Conexibacter*, *Gaiella*, *Candidatus Gaiellasilicea*, *Anaeromyxobacter*, and *Mycobacterium*. NO_3_^–^-N was positively correlated with *Gemmatimonas* and *Rhodanobacter* and negatively correlated with *Methyloceanibacter*, *Conexibacter*, *Gaiella*, *Candidatus Gaiellasilicea*, *Kouleothrix*, *Anaeromyxobacter*, *Mycobacterium*, and *Singulisphaera*. Urease (S-UE) was negatively correlated with *Gemmatimonas*. Dehydrogenase (S-DHA) was positively correlated with *Singulisphaera*. Nitrite reductase (S-NIR) was positively correlated with *Methyloceanibacter* and *Kouleothrix* (Figure 4E).

### 3.4 Changes in xenobiotic biodegradation pathways

**FIGURE 5:**
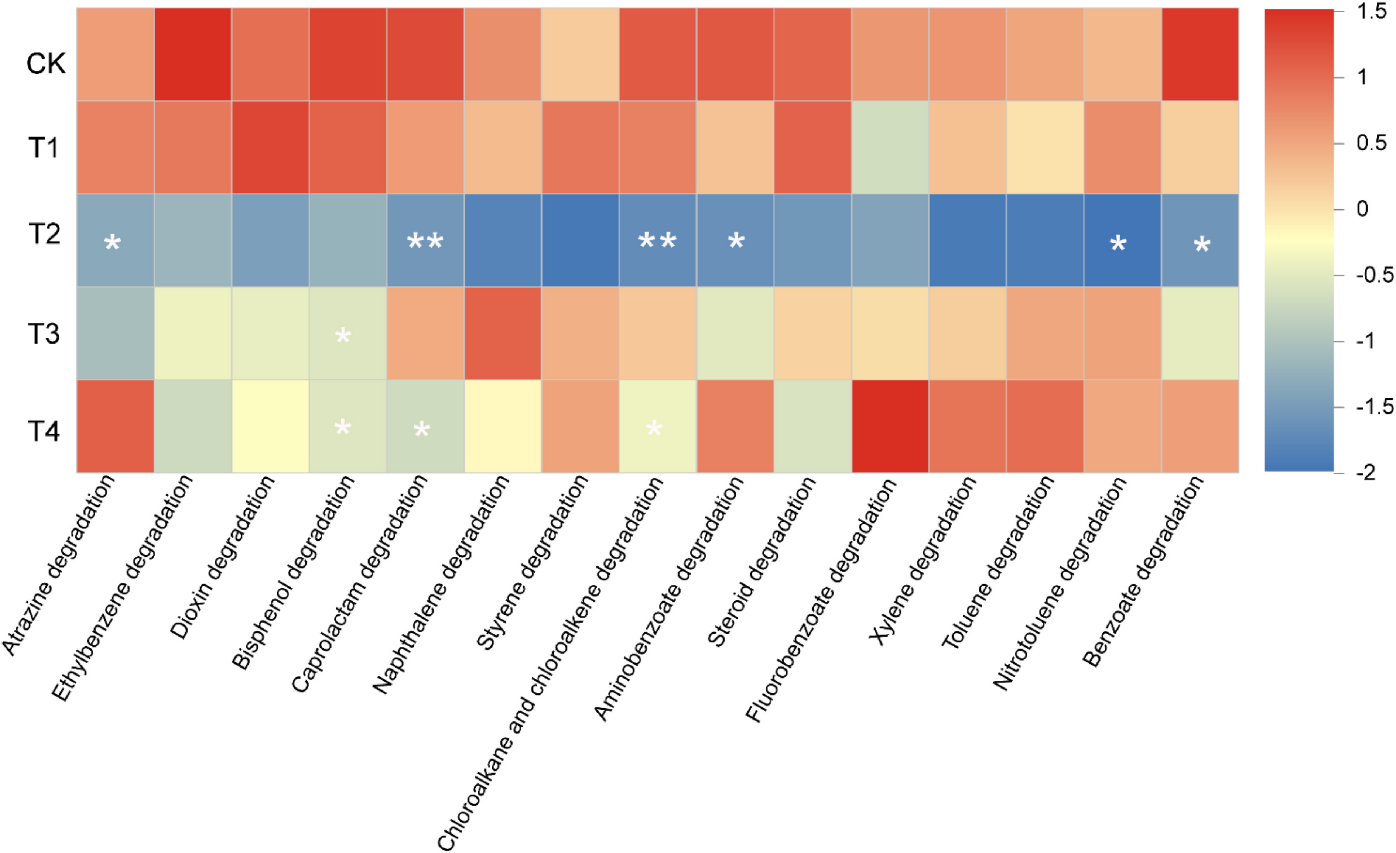
Heatmap of xenobiotic biodegradation (*P < 0.05, **P < 0.01 vs CK).

Metagenomic analysis identified a total of 15 xenobiotic degradation pathways (Figure 5). The results indicated that pesticide treatments inhibited the degradation capacity of soil microorganisms, with the extent of inhibition varying by pesticide type. First, all treatments collectively suppressed six degradation pathways, including Ethylbenzene degradation, Bisphenol degradation, Caprolactam degradation, Chloroalkane and chloroalkene degradation, Aminobenzoate degradation, and Benzoate degradation.

The suppressive effects of the pesticide treatments on degradation pathways varied in scope. The T2 treatment exhibited the broadest impact, inhibiting all 15 pathways. Among these, six pathways—Atrazine degradation, Caprolactam degradation, Chloroalkane and chloroalkene degradation, Aminobenzoate degradation, Nitrotoluene degradation, and Benzoate degradation—showed significant differences (*P* < 0.05). The T1 treatment suppressed 10 pathways. T3 suppressed 11 pathways, among which Bisphenol degradation was significantly decreased (*P* < 0.05). The T4 treatment inhibited 9 pathways, with significant suppression observed in Bisphenol degradation and Caprolactam degradation (*P* < 0.05).

In summary, pesticide treatments generally suppressed the microbial degradation capacity for exogenous substances. The T2 treatment had the most extensive and significant impact, while certain pathways, such as Bisphenol degradation, were significantly inhibited across multiple treatments.

### 3.5 Alterations in nitrogen transformation pathways

**FIGURE 6:**
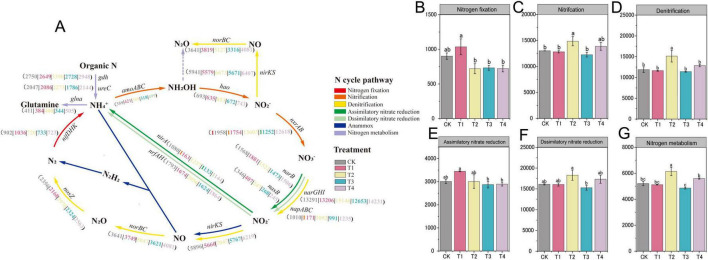
**(A)** Nitrogen cycling pathways. **(B–G)**; bar chart of nitrogen cycle pathways: **(B)** nitrogen fixation, **(C)** nitrification, **(D)** denitrification, **(E)** assimilatory nitrate reduction, **(F)** dissimilatory nitrate reduction, **(G)** nitrogen metabolism. The superscript letters ‘a–c’ indicate statistically significant differences (*p* < 0.05) among different treatments as determined by Duncan’s multiple range test.

To evaluate the effects of the four pesticides on soil nitrogen transformation processes, we analyzed the key functional genes associated with the nitrogen cycle (Figure 6A). The genes involved included those for nitrogen fixation *(nifD*, *nifH*, *nifK*), nitrification *(amoA*, *amoB*, *amoC*, *hao*, *nxrA*, *nxrB*), denitrification (*nirK*, *nirS*, *norB*, *norC*, *nosZ*), assimilatory nitrate reduction to ammonium (ANRA: *nasB*, *narB*, *nirA*), dissimilatory nitrate reduction to ammonium (DNRA: *narG*, *narH*, *narI*, *napA*, *napB*, *napC*, *nrfA*, *nrfH*), and organic nitrogen metabolism (*ureC*, *gdh*, *glnA*). The results revealed that different pesticide treatments significantly affected nitrogen cycling pathways: The T1 treatment enhanced the nitrogen fixation pathway. The T2 treatment significantly strengthened the nitrification, denitrification, and nitrogen metabolism pathways, with levels reaching 1.14 times, 1.27 times, and 1.18 times that of the control, respectively (Figures 6C, D). The T3 and T4 treatments showed no significant impact on the soil nitrogen cycle processes (Figures 6B–G).

### 3.6 Association between soil nitrogen cycle genes and microorganisms

**FIGURE 7:**
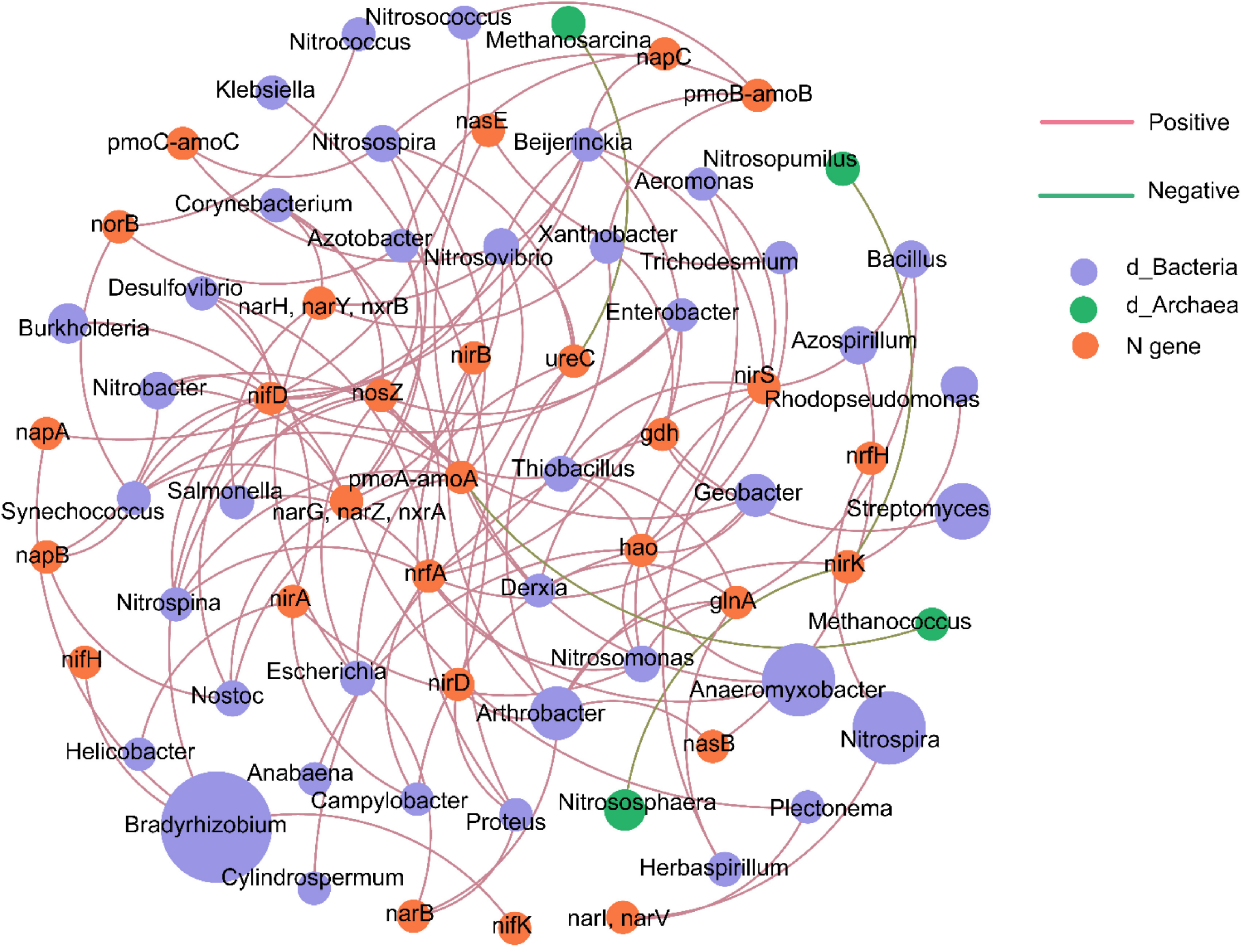
Co-occurrence network between nitrogen cycle genes and microbial genera. Edges represent correlations, with green and pink indicating positive and negative relationships, respectively. Nodes are colored according to their classification as different nitrogen cycle genes or microbial phyla.

The co-occurrence network analysis revealed 70 nodes in total, comprising 27 nitrogen cycle gene nodes and 43 microbial genus nodes (Figure 7). Correlation analysis indicated that the abundance of nitrogen fixation genes (nifD, nifH, nifK) was significantly and positively correlated with several microbial genera, including *Bradyrhizobium*, *Nitrobacter*, *Nostoc*, *Desulfovibrio*, *Nitrospina*, *Nitrosococcus*, *Synechococcus*, and *Beijerinckia*. The abundance of key nitrification genes (*hao*, *nxrA*, *nxrB*) was positively correlated with a range of genera, such as *Anaeromyxobacter*, *Geobacter*, *Nitrosomonas*, *Thiobacillus*, *Aeromonas*, *Anabaena*, *Trichodesmium*, *Campylobacter*, *Burkholderia*, *Nitrobacter*, *Nitrosovibrio*, *Escherichia*, and *Salmonella*.

## 4 Discussion

### 4.1 Effects of pesticides on soil nitrogen and enzyme activities

Pesticide treatments differentially affected the transformation of soil nitrogen forms and key enzyme activities. Prothioconazole and Pyrisoxazole treatments significantly increased the soil NH_4_^+^-N content, likely from the inhibition and promotion of specific microbial populations that altering nitrogen transformation pathways (Castaldi and Smith, 1998). Similarly, Rose et al. (2018) also found that the herbicides Metsulfuron-methyl and 2,4-dichlorophenoxyacetic acid promoted NH_4_^+^-N content. Notably, pyrisoxazole simultaneously enhanced both NH_4_^+^-N and NO_3_^–^-N, presumably by partially activating nitrification. This aligns with the NO_3_^–^-N increase reported in hexaconazole-treated soils (Ju et al., 2017). Chen et al. (2001) suggested that the accumulation of both NH_4_^+^-N and NO_3_^–^-N by captan might relate to a lower nitrification rate coupled with higher urease activity.

Regarding soil enzyme activity, the kasugamycin combined with *Paenibacillus polymyxa* and cyclobutrifluram treatments significantly enhanced urease activity, while pyrisoxazole markedly inhibited this enzyme’s activity. Satapute et al. (2019) reported that propiconazole application to soil initially enhanced urease activity, but significantly inhibited it with increasing application rates, suggesting that these changes are influenced by both pesticide concentration and treatment duration. Bacmaga et al. (2020) found that tebuconazole inhibited urease activity by 15.6% to 59.9% in a dose-dependent manner, an effect they attribute to the fungicide’s toxicity toward the soil microbial community and its consequent impact on microbial enzyme secretion. Singh (2005) found that triadimefon application in soils reduced dehydrogenase activity by 70% and 50%, respectively, which might delay the degradation process of the fungicide in the soil. The changes in nitrogen and enzyme activities suggest that the metabolic activity and community structure of soil microorganisms may have altered.

### 4.2 Effects of pesticides on microbial diversity and community structure

Pesticide application alters the soil environment and influences the structure of microbial communities. In this study, Pseudomonadota, Actinobacteria, and Chloroflexi were the dominant phyla, contributing, respectively, to biocontrol, pathogen suppression, and soil aggregation (Ren et al., 2024; Barka et al., 2015; Luan et al., 2020; Wei et al., 2022). Additionally, Myxococcota, known for its predatory characteristics, was significantly enriched under the T2 treatment, potentially due to the release of biomass resources following pesticide-induced microbial mortality (Muñoz-Dorado et al., 2016). The widespread enrichment of Gemmatimonadota under both T2 and T4 treatments suggests its high tolerance to environmental stress (Hao et al., 2025; Yuan et al., 2024).

The T2 treatment notably enriched *Gemmatimonas*. Research indicates that *Gemmatimonas* can enhance soil nitrogen content (Zhang L. et al., 2025) and promote organic nitrogen mineralization (Hui et al., 2020), which aligns with the observed rise in NH_4_^+^-N content under the T2 treatment. Concurrently, this study found a negative correlation between *Gemmatimonas* and urease activity, indicating that urea hydrolysis was not the dominant pathway for nitrogen transformation under this treatment. The T2 treatment likely first enriched functional genera such as *Gemmatimonas*, driving intense organic nitrogen mineralization, thereby substantially increasing soil NH_4_^+^-N content. Subsequently, this NH_4_^+^-N was converted into NO_3_^–^-N through nitrification, facilitated by the enrichment of ammonia-oxidizing bacteria such as *Nitrosospira* and *Nitrosovibrio*, ultimately resulting in the simultaneous accumulation of both nitrogen forms (Brochado et al., 2023).

In contrast, the T4 treatment enriched multiple key nitrifying genera, including the ammonia-oxidizing genus *Nitrosovibrio* and the nitrite-oxidizing genera *Nitrobacter* and *Nitrospira* (Ayiti et al., 2022). These taxa participate in ammonia and nitrite oxidation processes, forming an efficient nitrification chain that rapidly converts NH_4_^+^-N into NO_3_^–^-N (Matsuba et al., 2003; Sánchez et al., 2004).

### 4.3 Effects of pesticides on soil xenobiotic degradation capacity

Soil microbial communities degrade various xenobiotic pollutants to maintain soil health. Studies confirm the crucial role of specific functional microorganisms. For instance, Zhang et al. (2020) demonstrated that the bacterial strain *Brevundimonas naejangsanensis* J3 can degrade the harmful xenobiotic dimethachlon. Similarly, Wang X. et al. (2025) found that *Bacillus subtilis* ZW plays a significant role in the bioremediation of p-cresol and other aromatic compounds.

In this study, metagenomic data revealed that pesticide treatments exhibited inhibitory trends across multiple xenobiotic degradation pathways. Concurrently, fungicide root irrigation significantly inhibited the overall metabolic activity of microorganisms, also reflecting potential disruption to the microbial community structure and functional diversity (Xu et al., 2020). Therefore, we speculate that the four fungicides may have interfered with or weakened the activity of key functional microorganisms, leading to concurrent functional impairments in several associated degradation pathways. This broad attenuation of soil detoxification potential, even if mostly not statistically significant, may further increase the potential ecological risks of persistent existence, accumulation, and migration of residual pollutants in the environment.

### 4.4 Effects of pesticides on soil nitrogen transformation pathways

Metagenomic analysis reveals how pesticides regulate nitrogen transformation pathways at the functional gene level. In the T1 treatment, NH_4_^+^-N accumulation was primarily linked to upregulation of the ANRA pathway gene *nirA*. Prothioconazole likely inhibited nitrifying microorganisms or altered microbial community structure, thereby relieving the potential suppression on assimilatory nitrate reduction and ultimately inducing *nirA* expression, which promoted NH_4_^+^-N accumulation (Rahman et al., 2021; Zhang et al., 2021). In contrast, T2 treatment accumulates NH_4_^+^-N by reshaping the nitrogen cycling pathways. It significantly suppressed the urease activity, thereby suppressing the urea hydrolysis pathway for NH_4_^+^-N production, but activated organic nitrogen mineralization pathway via microbial necromass (reflected by *gdh upregulation*). This pathway remodeling led to robust mineralization that outweighed the suppressed urea hydrolysis, becoming the main driver of NH_4_^+^-N accumulation. This may result from substantial microbial mortality caused by pyrisoxazole (Bünemann et al., 2006), which rapidly released cellular nitrogen pools via mineralization (Jenkinson and Parry, 1989). This reveals the resilience of the microbial community in maintaining nitrogen transformation functionality through compensatory responses in functional pathways (Allison and Martiny, 2008). Concurrently, enhanced nitrification gene (*nxrAB*) expression promoted NO_3_^–^-N accumulation. In T4, NO_3_^–^-N increase was primarily due to *nxrAB* upregulation.

Pesticides can affect the transformation and accumulation of nitrate and NH_4_^+^-N in soil by regulating the expression of key functional genes involved in the nitrogen cycle within the soil microbial community. This finding aligns with several previous studies: Cao et al. (2023) found that the herbicide mesosulfuron-methyl could influence nitrification and denitrification processes by altering the abundance of soil nitrogen cycle functional genes, thereby regulating NO_3_^–^-N and NH_4_^+^-N content. Lyu et al. (2024) reported that the herbicide Acetochlor inhibited NO_3_^–^-N transformation and reduced potential nitrification and denitrification rates, mechanisms associated with changes in enzyme activity and microbial communities. Du et al. (2018) also observed that the application of the herbicide mesotrione induced changes in soil microbial community composition, subsequently affecting NO_3_^–^-N and NH_4_^+^-N content.

### 4.5 Correlation between nitrogen cycle genes and microorganisms

By constructing a correlation network between nitrogen cycling genes and microbial genera, this study revealed the key microbial taxa driving functional changes in soil nitrogen cycling under pesticide stress. Notably, the network revealed both established and unexpected links. Expected correlations included positive links between nitrification genes (*hao*, *nxrAB*) and known nitrifying microorganisms such as *Nitrobacter* and *Nitrosovibrio* (Farges et al., 2012), confirming nitrification as the source of nitrate increases in T2 and T4. We also observed atypical patterns: nitrogen-fixing genes correlated not only with known diazotrophs (e.g., *Bradyrhizobium*, *Nostoc*), but also with the nitrifier *Nitrobacter*. These unusual connections may reflect functional reorganization of microbes under pesticide stress or new interspecies partnerships (Wertz et al., 2012; Dobrojan et al., 2016; Zhong et al., 2024).

Moreover, the associations between microbial genera and N-cycle genes highlights how microbial community shifts drive nitrogen cycling, aligning with reports on *Massilia* and *Arthrobacter* by Shi et al. (2021). In conclusion, the fungicide treatments not only affected the abundance of nitrogen cycle genes but also reconfigured the functional network of the nitrogen cycle by altering the microbial community structure.

## 5 Conclusion

Pesticide application differentially influenced soil microbial community structure and nitrogen cycle functioning. The most substantial structural shifts were observed in the T2 and T4 treatments: T2 was notably enriched with *Gemmatimonas*, *Nitrosospira*, *Nitrosovibrio*, and *Rhodanobacter*, while T4 was enriched with *Nitrobacter*, *Nitrospira*, *Nitrosovibrio*, and *Rhodanobacter*. Furthermore, the capacity for xenobiotic degradation was generally suppressed by the four pesticide treatments, with the T2 treatment exhibiting the broadest range of suppression, indicating a potential risk to the self-purification capacity of the soil ecosystem. Regarding the nitrogen cycle, the T1, T2, and T4 treatments influenced the transformation and accumulation of NH_4_^+^-N and NO_3_^–^-N by regulating the expression of key nitrogen cycle functional genes in soil microorganisms. Moreover, the T2 treatment enhanced the expression of the *gdh* gene and nitrification genes, collectively promoting the accumulation of NH_4_^+^-N and NO_3_^–^-N. This study provides a theoretical basis for the scientific assessment of the soil ecological risks of these four pesticides and their rational application. Our findings indicate that pesticide-induced changes in microbial community structure likely drive differences in nitrogen transformation by modulating the abundance and expression of functional genes involved in the nitrogen cycle.

## Data Availability

The datasets presented in this study can be found in online repositories. The names of the repository/repositories and accession number(s) can be found below: https://www.ncbi.nlm.nih.gov/, PRJNA1346056.

## References

[B1] Allison S. D. Martiny J. B. H. (2008). Resistance, resilience, and redundancy in microbial communities. *Proc. Natl. Acad. Sci. U S A.* 105 11512–11519. 10.1073/pnas.0801925105 18695234 PMC2556421

[B2] Aßhauer K. P. Wemheuer B. Daniel R. Meinicke P. (2015). Tax4Fun: predicting functional profiles from metagenomic 16S rRNA data. *Bioinformatics* 31 2882–2884. 10.1093/bioinformatics/btv287 25957349 PMC4547618

[B3] Astm International. (2010). *Standard test methods for laboratory determination of water (moisture) content of soil and rock by Mass. ASTM D2216-10.* West Conshohocken, PA: ASTM International.

[B4] Ayiti O. E. Ayangbenro A. S. Babalola O. O. (2022). 16S amplicon sequencing of nitrifying bacteria and archaea inhabiting maize rhizosphere and the influencing environmental factors. *Agriculture* 12:1328. 10.3390/agriculture12091328

[B5] Bacmaga M. Wyszkowska J. Kucharski J. (2020). Response of soil microorganisms and enzymes to the foliar application of Helicur 250 EW fungicide on Hordeum vulgare L. *Chemosphere* 242:125163. 10.1016/j.chemosphere.2019.125163 31677518

[B6] Barka E. A. Vatsa P. Sanchez L. Gaveau-Vaillant N. Jacquard C. Klenk H.-P. (2015). Taxonomy, physiology, and natural products of Actinobacteria. *Microbiol. Mol. Biol. Rev.* 80 1–43. 10.1128/mmbr.00019-15 26609051 PMC4711186

[B7] Brochado M. G. S. da Silva L. B. X. Lima A. da C. Guidi Y. M. Mendes K. F. (2023). Herbicides versus nitrogen cycle: Assessing the trade-offs for soil integrity and crop yield-an in-depth systematic review. *Nitrogen* 4 296–310. 10.3390/nitrogen4030022

[B8] Buchfink B. Xie C. Huson D. H. (2015). Fast and sensitive protein alignment using DIAMOND. *Nat. Methods* 12 59–60. 10.1038/nmeth.3176 25402007

[B9] Bünemann E. K. Schwenke G. D. Van Zwieten L. (2006). Impact of agricultural inputs on soil organisms-a review. *Aust. J. Soil Res.* 44 379–406. 10.1071/SR05125

[B10] Cao J. L. Zhang Y. Dai G. C. Cui K. Wu X. H. Qin F. X. (2023). The long-acting herbicide mesosulfuron-methyl inhibits soil microbial community assembly mediating nitrogen cycling. *J. Hazard. Mater.* 443:130293. 10.1016/j.jhazmat.2022.130293 36444049

[B11] Castaldi S. Smith K. A. (1998). Effect of cycloheximide an N_2_O and NO_3_^–^ production in a forest and an agricultural soil. *Biol. Fertil. Soils* 27 27–34. 10.1007/s003740050395

[B12] Chao A. (1984). Nonparametric estimation of the number of classes in a population. *Scand. J. Stat.* 11 265–270.

[B13] Chen S. K. Edwards C. A. Subler S. (2001). A microcosm approach for evaluating the effects of the fungicides benomyl and captan on soil ecological processes and plant growth. *Appl. Soil Ecol.* 18 69–82. 10.1016/S0929-1393(01)00135-4

[B14] Chen Y. Zhang F. Huang B. Wang J. Huang H. Song Z. (2022). Effects of oxathiapiprolin on the structure, diversity and function of soil fungal community. *Toxics* 10:548. 10.3390/toxics10090548 36136513 PMC9504812

[B15] Cycoń M. Mrozik A. Piotrowska-Seget Z. (2019). Antibiotics in the soil environment-degradation and their impact on microbial activity and diversity. *Front. Microbiol.* 10:338. 10.3389/fmicb.2019.00338 30906284 PMC6418018

[B16] Dobrojan S. Salaru V. Stratulat I. Dobrojan G. (2016). Study of the biological nitrogen fixation process to combination of some blue-green algae from the genus Nostoc. *Sci. Paper Ser. A Agron.* 59 59–62.

[B17] Du P. Wu X. Xu J. Dong F. Liu X. Zheng Y. (2018). Effects of trifluralin on the soil microbial community and functional groups involved in nitrogen cycling. *J. Hazard. Mater.* 353 204–213. 10.1016/j.jhazmat.2018.04.012 29674095

[B18] Ezeokoli O. T. Bezuidenhout C. C. Maboeta M. S. Khasa D. P. Adeleke R. A. (2020). Structural and functional differentiation of bacterial communities in post-coal mining reclamation soils of South Africa: Bioindicators of soil ecosystem restoration. *Sci. Rep.* 10:1759. 10.1038/s41598-020-58576-5 32019965 PMC7000389

[B19] Farges B. Poughon L. Roriz D. Creuly C. Dussap C. G. Lasseur C. (2012). Axenic cultures of Nitrosomonas europaea and Nitrobacter winogradskyi in autotrophic conditions: a new protocol for kinetic studies. *Appl. Biochem. Biotechnol.* 167 1076–1091. 10.1007/s12010-012-9651-6 22451350

[B20] Faria M. Bertocco T. Barroso A. Carvalho M. Fonseca F. Matos C. D. (2023). A Comparison of analytical methods for the determination of soil pH: Case study on burned soils in northern portugal. *Soil Syst.* 6:227. 10.3390/fire6060227

[B21] Fu L. Niu B. Zhu Z. Wu S. Li W. (2012). CD-HIT: Accelerated for clustering the next-generation sequencing data. *Bioinformatics* 28 3150–3152. 10.1093/bioinformatics/bts565 23060610 PMC3516142

[B22] Gao Q. Ma J. Liu Q. Liao M. Xiao J. Jiang M. (2020). Effect of application method and formulation on prothioconazole residue behavior and mycotoxin contamination in wheat. *Sci. Total Environ*. 729:139019. 10.1016/j.scitotenv.2020.139019 32361459

[B23] Han L. Wang Y. Wang Y. Xu H. Liu M. Nie J. (2024). Pyraclostrobin repeated treatment altered the degradation behavior in soil and negatively affected soil bacterial communities and functions. *J. Hazard. Mater.* 485:136876. 10.1016/j.jhazmat.2024.136876 39694009

[B24] Hao R. Li H. Qin S. Chen W. Guo Q. Huang Y. (2025). Effect of drought stress on root metabolome and soil microbial characteristics for maize (Zea Mays L.) seedlings. *Curr. Microbiol.* 82:415. 10.1007/s00284-025-04395-8 40719853

[B25] Hou M. Zhu Y. Chen H. Wen Y. (2024). Chiral herbicide imazethapy influences plant-soil feedback on nitrogen metabolism by shaping rhizosphere microorganisms. *Environ. Sci. Pollut. Res. Int.* 31 18625–18635. 10.1007/s11356-024-32393-z 38351351

[B26] Huang B. Jia H. Han X. Gou J. Huang C. Wang J. (2021). Effects of biocontrol bacillus and fermentation bacteria additions on the microbial community, functions and antibiotic resistance genes of prickly ash seed oil meal-biochar compost. *Bioresour. Technol.* 340:125668. 10.1016/j.biortech.2021.125668 34339999

[B27] Hui C. Jiang H. Liu B. Wei R. Zhang Y. Zhang Q. (2020). Chitin degradation and the temporary response of bacterial chitinolytic communities to chitin amendment in soil under different fertilization regimes. *Sci. Total Environ.* 705:136003. 10.1016/j.scitotenv.2019.136003 31846813

[B28] Jenkinson D. S. Parry L. C. (1989). The nitrogen cycle in the broadbalk wheat experiment: A model for the turnover of nitrogen through the soil microbial biomass. *Soil Biol. Biochem.* 21 535–541. 10.1016/0038-0717(89)90127-2

[B29] Jia S. J. Cui M. Y. Chen L. Guo S. Y. Zhang H. Bai Z. Y. (2025). Soybean water monitoring and water demand prediction in arid region based on UAV multispectral data. *Agronomy* 15:88. 10.3390/agronomy15010088

[B30] Jiao B. Zhu Y. Xu J. Dong F. Wu X. Liu X. (2022). Identification and ecotoxicity prediction of pyrisoxazole transformation products formed in soil and water using an effective HRMS workflow. *J. Hazard. Mater.* 424:127223. 10.1016/j.jhazmat.2021.127223 34600378

[B31] Ju C. Xu J. Wu X. Dong F. Liu X. Tian C. (2017). Effects of hexaconazole application on soil microbes community and nitrogen transformations in paddy soils. *Sci. Total Environ.* 609 655–663. 10.1016/j.scitotenv.2017.07.146 28763662

[B32] Kong X. Wang S. Li J. Zhang K. Yin Y. Li Y. (2025). Kasugamycin and Validamycin differentially inhibit housefly larval growth through gut microbiota regulation. *Ecotoxicol. Environ. Saf*. 304:119098. 10.1016/j.ecoenv.2025.119098 41027194

[B33] Kuypers M. M. M. Marchant H. K. Kartal B. (2018). The microbial nitrogen-cycling network. *Nat. Rev. Microbiol.* 16 263–276. 10.1038/nrmicro.2018.9 29398704

[B34] Li D. Liu C. M. Luo R. Sadakane K. Lam T. W. (2015). MEGAHIT: An ultra-fast single-node solution for large and complex metagenomics assembly via succinct de Bruijn graph. *Bioinformatics* 31 1674–1676. 10.1093/bioinformatics/btv033 25609793

[B35] Lin H. Dong B. Hu J. (2017). Residue and intake risk assessment of prothioconazole and its metabolite prothioconazole-desthio in wheat field. *Environ. Monit. Assess*. 189:236. 10.1007/s10661-017-5943-1 28451958

[B36] Liu Y. Zheng Q. Niu L. Liu X. Shen S. Zhou Y. (2025). Control efficacy of 9 fungicides on tobacco Fusarium root rot. *Agrochemicals* 64 601–608. 10.16820/j.nyzz.2025.0811

[B37] Luan L. Liang C. Chen L. Wang H. Xu Q. Jiang Y. (2020). Coupling bacterial community assembly to microbial metabolism across soil profiles. *mSystems* 5:e00298-20. 10.1128/mSystems.00298-20 32518195 PMC7289589

[B38] Lyu C. Cui J. Jin F. Li X. Xu Y. (2024). Impacts of acetochlor on nitrogen-cycling-related microbial communities in riparian zone soils. *Water* 16:461. 10.3390/w16030461

[B39] Materon I. C. Palzkill T. (2023). Structural biology of MCR-1-mediated resistance to polymyxin antibiotics. *Curr. Opin. Struct. Biol.* 82:102647. 10.1016/j.sbi.2023.102647 37399693 PMC10527939

[B40] Matsuba D. Takazaki H. Sato Y. Takahashi R. Tokuyama T. Wakabayashi K. (2003). Susceptibility of ammonia-oxidizing bacteria to nitrification inhibitors. *Z. Naturforsch. C J. Biosci.* 58 282–287. 10.1515/znc-2003-3-424 12710742

[B41] Metch J. W. Burrows N. D. Murphy C. J. Pruden A. Vikesland P. J. (2018). Metagenomic analysis of microbial communities yields insight into impacts of nanoparticle design. *Nat. Nanotechnol.* 13 253–259. 10.1038/s41565-017-0029-3 29335567

[B42] Muñoz-Dorado J. Marcos-Torres F. J. García-Bravo E. Moraleda-Muñoz A. Pérez J. (2016). Myxobacteria: Moving, killing, feeding, and surviving together. *Front. Microbiol.* 7:781. 10.3389/fmicb.2016.00781 27303375 PMC4880591

[B43] Noguchi H. Park J. Takagi T. (2006). MetaGene: Prokaryotic gene finding from environmental genome shotgun sequences. *Nucleic Acids Res.* 34 5623–5630. 10.1093/nar/gkl723 17028096 PMC1636498

[B44] Page A. L. (1982). *Methods of soil analysis. Part 2. Chemical and microbiological properties.* Madison, WI: American Society of Agronomy.

[B45] Pal C. Bengtsson-Palme J. Rensing C. Kristiansson E. Larsson D. G. J. (2014). BacMet: Antibacterial biocide and metal resistance genes database. *Nucleic Acids Res.* 42 D737–D743. 10.1093/nar/gkt1252 24304895 PMC3965030

[B46] Pan X. Dong F. Xu J. Liu X. Chen Z. Zheng Y. (2016). Stereoselective analysis of novel chiral fungicide pyrisoxazole in cucumber, tomato and soil under different application methods with supercritical fluid chromatography/tandem mass spectrometry. *J. Hazard. Mater.* 311 115–124. 10.1016/j.jhazmat.2016.03.005 26970041

[B47] Qi P. Yuan Y. Wang Z. Wang X. Xu H. Zhang H. (2016). Use of liquid chromatography-quadrupole time-of-flight mass spectrometry for enantioselective separation and determination of pyrisoxazole in vegetables, strawberry and soil. *J. Chromatogr. A* 1449 62–70. 10.1016/j.chroma.2016.04.051 27133864

[B48] Rahman M. M. Khanom A. Biswas S. K. (2021). Effect of pesticides and chemical fertilizers on the nitrogen cycle and functional microbial communities in paddy soils: Bangladesh perspective. *Bull. Environ. Contam. Toxicol.* 106 243–249. 10.1007/s00128-020-03092-5 33452610

[B49] Ren J. Cao T. Zang X. Liu J. Yang D. (2024). Antifungal mechanisms and characteristics of *Pseudomonas fluorescens*: Promoting peanut growth and combating Fusarium oxysporum-induced root rot. *Plant Physiol. Biochem.* 216:109092. 10.1016/j.plaphy.2024.109092 39241626

[B50] Rose M. T. Ng E. L. Weng Z. Wood R. Rose T. J. Van Zwieten L. (2018). Minor effects of herbicides on microbial activity in agricultural soils are detected by N-transformation but not enzyme activity assays. *Eur. J. Soil Biol.* 87 72–79. 10.1016/j.ejsobi.2018.04.003

[B51] Saha U. K. Sonon L. Biswas B. K. (2018). A comparison of diffusion-conductimetric and distillation-titration methods in analyzing ammonium- and nitrate-nitrogen in the KCl-extracts of georgia soils. *Commun. Soil Sci. Plant Anal.* 49 63–75. 10.1080/00103624.2017.1421647

[B52] Sánchez O. Aspé E. Martí M. C. Roeckel M. (2004). The effect of sodium chloride on the two-step kinetics of the nitrifying process. *Water Environ. Res.* 76 73–80. 10.2175/106143004X141609 15058467

[B53] Satapute P. Kamble M. V. Adhikari S. S. Jogaiah S. (2019). Influence of triazole pesticides on tillage soil microbial populations and metabolic changes. *Sci. Total Environ.* 651 2334–2344. 10.1016/j.scitotenv.2018.10.099 30336423

[B54] Schloss P. D. Westcott S. L. Ryabin T. Hall J. R. Hartmann M. Hollister E. B. (2009). Introducing mothur: Open-source, platform-independent, community-supported software for describing and comparing microbial communities. *Appl. Environ. Microbiol.* 75 7537–7541. 10.1128/AEM.01541-09 19801464 PMC2786419

[B55] Shannon C. E. (1948). A mathematical theory of communication. *Bell Syst. Tech. J.* 27 379–423. 10.1002/j.1538-7305.1948.tb01338.x

[B56] Shi L. Zhang P. Chen Q. Yang C. Zhang D. Xu J. (2021). Selective influence of cyflumetofen in degradation and ecological risk assessment. *Land Degrad. Dev.* 32 4920–4932. 10.1002/ldr.4080

[B57] Singh N. (2005). Factors affecting triadimefon degradation in soils. *J. Agric. Food Chem.* 53 70–75. 10.1021/jf048884j 15631511

[B58] Tang Q. Wang P. Liu H. Jin D. Chen X. Zhu L. (2023). Effect of chlorantraniliprole on soil bacterial and fungal diversity and community structure. *Heliyon* 9:e13668. 10.1016/j.heliyon.2023.e13668 36852024 PMC9957708

[B59] Trivedi P. Leach J. E. Tringe S. G. Sa T. Singh B. K. (2020). Plant microbiome interactions: From community assembly to plant health. *Nat. Rev. Microbiol*. 18, 607–621. 10.1038/s41579-020-0412-1 32788714

[B60] Wang X. Liu S. Ding X. Zhang L. Lv X. Li J. (2025). Coexistence of diverse metabolic pathways promotes p-cresol biodegradation by Bacillus subtilis ZW. *Int. Biodeterior. Biodegrad.* 196:105933. 10.1016/j.ibiod.2024.105933

[B61] Wang Y. Huang B. Wang Y. Ju C. Wang X. Han L. (2025). Successive hexaconazole application altered the degradation behavior in soil and shifted microbial community and functional profiles. *Environ. Res.* 286:122820. 10.1016/j.envres.2025.122820 40935104

[B62] Wei H. Y. Li Y. Peng S. Y. Huang L. Zhang B. Li K. T. (2022). Promoting crop growth under stress conditions by exopolysaccharides: review and perspective. *Jiangsu J. Agric. Sci.* 38 1123–1134. 10.3969/j.issn.1000-4440.2022.04.032

[B63] Wertz S. Leigh A. K. K. Grayston S. J. (2012). Effects of long-term fertilization of forest soils on potential nitrification and on the abundance and community structure of ammonia oxidizers and nitrite oxidizers. *FEMS Microbiol. Ecol.* 79 142–154. 10.1111/j.1574-6941.2011.01204.x 22066501

[B64] Xu J. Li J. Zhao X. Liu Z. Xu H. Cao K. (2025). Impact of reduced chemical fertilizer and organic amendments on yield, nitrogen use efficiency, and soil microbial dynamics in chinese flowering cabbage. *Horticulturae* 11:859. 10.3390/horticulturae11070859

[B65] Xu N. Qu Q. Zhang Z. Yuan W. Cui H. Shen Y. (2020). Effects of residual S-metolachlor in soil on the phyllosphere microbial communities of wheat (Triticum aestivum L.). *Sci. Total Environ.* 748:141342. 10.1016/j.scitotenv.2020.141342 32818888

[B66] Yang X. W. Qi P. P. Wang X. Y. Wang Z. W. Sun Y. H. Wang L. D. (2017). Stereoselective analysis and degradation of pyrisoxazole in cabbage, pakchoi, and pepper by liquid chromatography tandem mass spectrometry. *J. Agric. Food Chem.* 65 10139–10146. 10.1021/acs.jafc.7b02877 28863259

[B67] Yuan A. Kumar S. D. Wang H. Wang S. Impa S. Wang H. (2024). Dynamic interplay among soil nutrients, rhizosphere metabolites, and microbes shape drought and heat stress responses in summer maize. *Soil Biol. Biochem.* 191:109357. 10.1016/j.soilbio.2024.109357

[B68] Zhai W. Zhang L. Liu H. Zhang C. Liu D. Wang P. (2022). Enantioselective degradation of prothioconazole in soil and the impacts on the enzymes and microbial community. *Sci. Total Environ.* 824:153658. 10.1016/j.scitotenv.2022.153658 35151744

[B69] Zhang C. Li J. H. An H. M. Wu X. M. Wu Y. Y. Long Y. H. (2020). Enhanced elimination of dimethachlon from soils using a novel strain Brevundimonas naejangsanensis J3. *J. Environ. Manage.* 255:109848. 10.1016/j.jenvman.2019.109848 31756580

[B70] Zhang G. Li J. Cui B. Xue R. Liu J. Han S. (2025). Identification of tobacco fusarium root rot in Rizhao, Shandong Province and screening of its control agents. *Jiangsu Agric. Sci.* 2025 133–138. 10.15889/j.issn.1002-1302.2025.10.017

[B71] Zhang L. Yin J. Ji W. Zhao J. Xin Z. Rao D. (2025). Long-term maize-soybean crop rotation: Impacts on soybean yield, soil microbiota and nitrogen dynamics. *Front. Plant Sci.* 16:1658885. 10.3389/fpls.2025.1658885 41220775 PMC12598005

[B72] Zhang Y. Zhang J. Shi B. Li B. Du Z. Wang J. (2021). Effects of cloransulam-methyl and diclosulam on soil nitrogen and carbon cycle-related microorganisms. *J. Hazard. Mater.* 418:126395. 10.1016/j.jhazmat.2021.126395 34329028

[B73] Zhang Z. Q. Han R. H. Wang S. Z. Wang L. X. Yang X. X. Guo D. (2024). Field efficacy of cyclobutrifluram 50% FS against sweet potato stem nematode disease. *Agrochemicals* 63 209–211. 10.16820/j.nyzz.2024.0310

[B74] Zhong C. Hu G. Hu C. Xu C. Zhang Z. Ning K. (2024). Comparative genomics analysis reveals genetic characteristics and nitrogen fixation profile of Bradyrhizobium. *iScience* 27:108948. 10.1016/j.isci.2024.108948 38322985 PMC10845061

